# How patients understand depression associated with chronic physical disease – a systematic review

**DOI:** 10.1186/1471-2296-13-41

**Published:** 2012-05-28

**Authors:** Sarah L Alderson, Robbie Foy, Liz Glidewell, Kate McLintock, Allan House

**Affiliations:** 1 Institute of Health Sciences, University of Leeds, Leeds, UK

**Keywords:** Depression, Comprehension, Primary health care, Chronic disease, Review, Systematic

## Abstract

**Background:**

Clinicians are encouraged to screen people with chronic physical illness for depression. Screening alone may not improve outcomes, especially if the process is incompatible with patient beliefs. The aim of this research is to understand people’s beliefs about depression, particularly in the presence of chronic physical disease.

**Methods:**

A mixed method systematic review involving a thematic analysis of qualitative studies and quantitative studies of beliefs held by people with current depressive symptoms.

MEDLINE, EMBASE, PSYCHINFO, CINAHL, BIOSIS, Web of Science, The Cochrane Library, UKCRN portfolio, National Research Register Archive, Clinicaltrials.gov and OpenSIGLE were searched from database inception to 31^st^ December 2010.

A narrative synthesis of qualitative and quantitative data, based initially upon illness representations and extended to include other themes not compatible with that framework.

**Results:**

A range of clinically relevant beliefs was identified from 65 studies including the difficulty in labeling depression, complex causal factors instead of the biological model, the roles of different treatments and negative views about the consequences of depression. We found other important themes less related to ideas about illness: the existence of a self-sustaining ‘depression spiral’; depression as an existential state; the ambiguous status of suicidal thinking; and the role of stigma and blame in depression.

**Conclusions:**

Approaches to detection of depression in physical illness need to be receptive to the range of beliefs held by patients. Patient beliefs have implications for engagement with depression screening.

## Background

Clinicians are often encouraged to identify emotional problems in patients with physical disorders. For example, guidance from the UK National Institute for Health and Clinical Excellence (NICE) states that “screening should be undertaken in primary care …for depression in high-risk groups” [1]. Up to a quarter of people with diabetes or coronary heart disease have depression [2,3] which is associated with poorer outcomes [4]. Policy initiatives in the UK have included financial incentives for general practitioners to screen all patients with coronary heart disease or diabetes [5] and expanded access to psychological services for people with long-term physical conditions [6]. Over 92% of eligible patients were screened in 2009–10 [7]. However, it is uncertain whether incentivising screening alone has improved patient outcomes [8].

Three conditions need to be satisfied for screening to improve outcomes: engagement of primary care staff with the screening process [9]; a systematic approach to patient management following detection [10-12], and patient engagement with the screening process. This review concentrates on the third condition which is relatively neglected in the previously published literature. Consultation models emphasize the importance of understanding patient perspectives in clinical care [13]. This may be particularly challenging in states such as depression which lack clear cut diagnostic boundaries, and in the context of depression screening in physical illness which usually takes place at chronic disease clinics, or opportunistically during other consultations, where time to explore patient beliefs is often limited. Beliefs will also influence the subsequent management of depression including adherence to anti-depressant medication regimes and referral to specialist mental health services [14-16].

We conducted a mixed methods systematic review of studies of people with current depressive symptoms, which investigated their beliefs about those symptoms.

## Methods

### Search strategy

**Figure 1:**
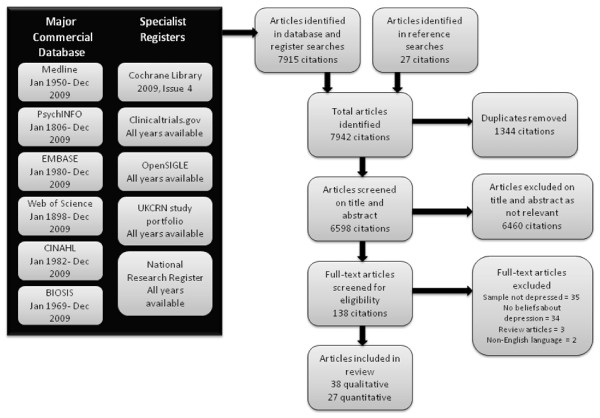
PRISMA chart of search strategy and identification of publications included in the review.

The review protocol can be requested from the study authors. We sought English-language studies of adults with current depressive symptoms that reported beliefs about depression. We systematically searched for articles and included studies of beliefs associated with chronic physical illness in stage 1 and then systematically searched for studies that included all depression beliefs in stage 2. Searches were limited to primary care where that was possible using the database search terms. We excluded non-English language studies and studies that assessed subjects without current depression or, explored beliefs about other mental health disorders (including anxiety, post-natal depression or bipolar disorder). Figure1 detail our search methods [17]. Appendix 1 details the search terms applied. We (SA) also reviewed reference lists of all included studies.

Initial screening of titles and abstracts, with exclusion of those that were obviously not related to depression beliefs, was undertaken by one reviewer (SA) with 18% (913) of studies reviewed by a second reviewer (KM). Full-text articles were assessed in detail by two reviewers (SA and KM) for all potentially eligible studies. All disagreements between reviewers were resolved by discussion.

### Data extraction and quality assessment

Data on study participants, methods and findings were abstracted from included studies using a standardised form specifically developed for this review. The findings of qualitative studies were entered verbatim into NVivo8, a qualitative data analysis software package. We assessed study quality using established criteria [18]. Authors were contacted for further information as required.

### Data synthesis

We conducted a narrative synthesis [19]. This approach to the synthesis of evidence relies primarily on the use of words and text to summarise and explain the findings of multiple studies. It is especially suited to a study like ours in which there is wide variation in study type included. Stage one involved a thematic and content analysis of the qualitative data. We initially categorised beliefs about depression using Leventhal’s Illness Representations [20], a framework for characterising patients’ beliefs about illnesses [21-23].

The illness representation includes five main categories of belief: identity (beliefs concerning label and associated symptoms), cause (factors and conditions believed to have caused a condition), timeline (acute, cyclical or chronic), consequences (expected effects on physical, social and psychological well-being) and the control and/or cure (to what extent treatment and behaviours will help), along with a parallel emotional representation. We also identified beliefs not adequately captured by the framework and developed new themes which were agreed by consensus. The coding of themes was checked for 10% of studies by a second researcher.

Reviews of the quantitative findings were mapped onto the framework derived from the qualitative literature. For example, the finding that 68% of participants in one study felt that having depression changed the way they viewed themselves [24] supported the theme of existential & self.

In stage two we assessed the robustness of the synthesis by appraising the contribution of weak studies to review findings. Quality was assessed using criteria appropriate to study design [18]. Studies were assigned a score and topics based upon weak studies only were not included in the final analysis.

The final stage involved integrating the findings from the preceding stages into overarching conclusions.

#### Ethical approval

This project did not require ethical approval.

## Results

We identified 7942 abstracts, of which 64 individual studies from 65 reports were included (Figure1). Table1 summarizes all included studies. Studies ranged widely in terms of questions addressed and methods used with 37 studies using qualitative interviews and 27 using self-administered questionnaires. The majority of the studies took place in the UK or the United States. Less than half (45%) applied theoretical frameworks to collect or analyze data, with Leventhal’s Illness Representations being the most commonly used in both qualitative and quantitative studies. Beliefs about depression associated with chronic physical diseases were identified specifically in only two qualitative studies; however participants in other studies referred to physical ill health in their beliefs. We therefore addressed all beliefs about depression within a single synthetic review. No studies were excluded from the review because of poor quality.

**Table 1:** Table of included Studies

**Study & Year of Publication**	**Country**	**Gender**	**Ethnicity**	**Setting**	**Research Approach**	**Theoretical Framework**	**Depression status**	**Quality Assessment**	**Aim**
ADDIS 1995 [25]	USA	Female > Male	not given	not given	Cross-sectional Survey	Reasons for Depression	Diagnostic Interview	C	To develop the Reasons For Depression questionnaire and measure its internal consistency and validity
ADDIS 1996 [26]	USA	not given	not given	Primary Care & Community	Cross-sectional Survey	None	Diagnostic Interview	C	To examine the relationships between clients reasons for depression and the outcome of treatment
ALLEN 1998 [27]	UK	Male = Female	not given	Secondary Care	Cross-sectional Survey	None	Screening Test	B	To examine the presence of depressive symptoms as well as attitudes to and knowledge of depression in a group of physically ill inpatients
AL-SAFFAR 2003 [28]	Kuwait	Male > Female	Arabic	Secondary Care	Cross-sectional Survey	Health Belief Model	Diagnostic Interview	B	To determine whether underlying attitudes and health beliefs of patients were affecting their decision to take their medication as prescribed
BACKENSTRASS 2007 [29]	Germany	Female > Male	not given	Primary Care	Semi-structured Interview	none	Diagnostic Interview	B	GP and sub-threshold depression patients views on diagnosis & treatment
BADGER 2007b [30]	UK	Female > Male	not given	Primary Care	Semi-structured Interview	none	Medical Records	B	Attitudes towards and use of self-chosen treatment in patients prescribed antidepressants
BANN 2004 [31]	USA	Female > Male	Mixed	Secondary Care	Cross-sectional Survey	Explanatory Model	Screening Test	C	To evaluate the psychometric properties of the EMD instrument
BOGNER 2008 [32]	USA	Female > Male	Mixed	Primary Care	Semi-structured Interview	none	Screening Test	A	Older patients’ perspectives on the relationship of heart disease to depression
BROWN 2001 [24]	USA	Female > Male	Mixed	Primary Care	Cross-sectional Survey	CS-SRM IR	Screening Test	C	To determine whether primary care patients’ personal illness cognitions for depression are associated with depression coping strategies and treatment related behavior
BROWN 2005 [33]	USA	Female > Male	White	Primary Care	Cross-sectional Survey	CS-SRM IR	Medical Records	B	To describe beliefs about antidepressants, examine the factor structure of the BMQ
BROWN 2007 [34]	USA	Female > Male	White	Primary Care	Cross-sectional Survey	CS-SRM IR	Medical Records	B	to describe personal illness models for depression and the relationship with functional disability
BURROUGHS 2006 [35]	UK	Both	not given	Primary Care	Semi-structured Interview	none	Screening Test	B	Primary care professionals ideas about depression in elderly and elderly views on depression as a problem & help-seeking
CABASSA 2008 [36]	USA	Female > Male	Hispanic	Primary Care	Cross-sectional Survey	CS-SRM IR	Screening Test	C	The aim of the present study is to conduct a confirmatory factor analysis (CFA) of the IPQR adapted for a clinical sample of depressed low-income Latinos served in primary care.
CAPE 1999 [37]	UK	Female > Male	not given	Primary Care	Semi-structured Interview	none	Screening Test	C	Patients’ reasons for not discussing emotional problems with GP
CHAKRABORTY 2009 [38]	India	Male = Female	Indian	Secondary Care	Cross-sectional Survey	None	Diagnostic Interview	B	Attitudes and beliefs of patients of first episode depression towards antidepressant treatment and the relationship between beliefs and treatment adherence
COOPER 1998 [39]	USA	Female > Male	Mixed	Primary Care	Cross-sectional Survey	None	Diagnostic Interview	B	To compare the views of African-American and white adult primary care patients regarding the importance of various aspects of depression care
COOPER 2000 [40]	USA	Female > Male	Mixed	Primary Care	Cross-sectional Survey	None	Screening Test	B	To select items for inclusion in an instrument to measure attitudes towards depression care
COOPER 2003 [41]	USA	Female > Male	Mixed	Primary Care	Cross-sectional Survey	Theory of Reasoned Action	Diagnostic Interview	B	The objective of this study was to examine whether racial and ethnic differences exist in patient attitudes toward depression care.
COOPER-PATRICK 1997 [42]	USA	Female > Male	Mixed	Primary Care	Focus Group	none	Medical Records	B	Health professionals and patients’ identifying attitudes that influence help-seeking
CORNFORD 2007 [43]	UK	Female > Male	not given	Primary Care	Semi-structured Interview	none	Screening Test	A	Lay beliefs about depression symptoms and how they manage them
DANIELSSON 2009 [44]	Sweden	Equal	Swedish	Primary Care	Semi-structured Interview	Gender Theory	Medical Records	A	To explore how primary care patients experience & understand depression and the impact of gender in this process
DEJMAN 2008 [45]	Iran	Female	Middle-Eastern	Secondary Care	Semi-structured Interview	Explanatory Model	Medical Records & Screening Test	B	Explanatory models of help-seeking and coping with depression in Iranian women
EDLUND 2008 [46]	USA	Male > Female	Mixed	Primary Care	Randomised Controlled Trial	Health Belief Model	Screening Test	B	To assess the extent to which beliefs changed in the intervention and treatment as usual arms of the study
FORTUNE 2004 [47]	UK	Female	not given	not given	Qualitative Writing & Cross-sectional survey	CS-SRM IR	Screening Test	C	To compare the structure and content of peoples models of depression with those of a physical illness
GARFIELD 2003 [48]	UK	Female > Male	Mixed	Primary Care	Semi-structured Interview	none	Medical Records	A	To identify factors of importance to patients when beginning courses of antidepressant treatment
GASK 2003 [49]	UK	Female > Male	not given	Primary Care	Semi-structured Interview	none	Medical Records	B	To explore depressed patients’ perceptions of the quality of care from GP’s
GIVENS 2006 [50]	USA	Female > Male	not given	Primary Care	Semi-structured Interview	Explanatory Model	Diagnostic Interview	B	To understand why older people are adverse to using antidepressants
GIVENS 2007 [51]	USA	Female > Male	Mixed	Online	Cross-sectional Survey	None	Screening Test	B	To describe ethnic differences in attitudes toward depression
GREEN 2002 [52]	UK	Female	Chinese	Primary & Secondary Care & Community	Semi-structured Interview	none	Screening Test	B	To identify barriers to Chinese women accessing help for depression
GRIME 2003 [53]	UK	Female > Male	not given	Primary Care & Community	Semi-structured Interview	none	Medical Records or Self Diagnosis	B	To understand patients views and experiences of taking antidepressants
HEIFNER 1997 [54]	USA	Male	not given	not given	Semi-structured Interview	none	Medical Records	B	To explore the male experience of depression
KANGAS 2001 [55]	Finland	Female > Male	not given	Community	Semi-structured Interview	Narrative Reconstruction	Self Diagnosis	C	To discover how people explain the cause of their depression
KARASZ 2003 [56]	USA	Female > Male	Mixed	Primary Care	Semi-structured Interview	CS-SRM IR	Screening Test	B	To explore patients’ conceptual labels of depression and build a theoretical model linking these to attitudes to treatment
KARASZ 2006 [57]	USA	Female > Male	Hispanic	Primary Care	Semi-structured Interview	none	Screening Test	C	To investigate Hispanic patients’ perceptions of primary care treatments for depression
KARASZ 2008 [58]	USA	Female > Male	Hispanic	Primary Care	Semi-structured Interview	CS-SRM IR	Screening Test	B	To explore the heterogeneity of depression experience
KARASZ 2009 [59]	USA	Female > Male	not given	Primary Care	Semi-structured Interview	CS-SRM IR	Screening Test	A	To examine conceptual models of depression in different ethnic groups and focusing on the degree to which patients conceptual models matched a bio-psychiatric model
KARP 1994 [60]	UK	Female > Male	White British	Secondary Care	Semi-structured Interview	Illness Career	Medical Records	A	How those suffering from uni-polar depression perceive, interpret, and understand a life condition that often seems incoherent, fragmented and intractable
KELLY 2007 [61]	USA	Female > Male	not given	Primary Care	Cross-sectional Survey	CS-SRM IR	Medical Records	B	To examine the relationships between beliefs about depression and emotion
KIRK 2001 [62]	USA	Female	Mixed	Primary Care	Cross-sectional Survey	None	Diagnostic Interview	C	Assessed pre-existing attitudes to depression and its treatment in a population of economically disadvantaged women
KUYKEN 1992 [63]	UK	Female > Male	not given	Secondary Care	Semi-structured Interview & Cross-sectional Survey	none	Screening Test	C	To investigate beliefs and attitudes towards depression in patients’ and compare them to lay people and psychologists
LEWIS 1995 [64]	UK	not given	not given	Primary & Secondary Care & Community	Semi-structured Interview	none	Medical Records or Self Diagnosis	B	To investigate the experience of depression as a meaningful experience
LEYKIN 2007 [65]	USA	not given	not given	Secondary Care	Randomised Controlled Trial	Reasons for Depression	Diagnostic Interview	C	To look at relation between beliefs and outcomes of therapies
LOWE 2006 [66]	Germany	Female > Male	not given	Secondary Care	Semi-structured Interview	none	Diagnostic Interview	C	To investigate attitudes towards treatment approaches
MANBER 2003 [67]	USA	Female > Male	Mixed	Secondary Care	Cross-sectional Survey	CS-SRM IR	Diagnostic Interview	C	To develop the Perception of Depressive illness questionnaire
MARTIN 2007a [67]	Brazil	Female	Brazilian	Secondary Care	Ethnographic observation & Semi-structured Interview	none	Medical Records	B	To describe the perception of depression for women in Embu, Sao Paulo
MARTIN 2007b [68]	Brazil	Female	Brazilian	Secondary Care	Ethnographic observation & Semi-structured Interview	none	Medical Records	B	To assess the meaning of depression in women diagnosed with the disorder, and the context of care given by the psychiatrists
MAXWELL 2005 [69]	UK	Female	not given	Primary Care	Semi-structured Interview	none	Medical Records	A	To explore GP and patients’ accounts of recognizing and treating depression
NOLAN 2005 [70]	UK	Female > Male	not given	Primary Care	Semi-structured Interview	none	Medical Records	B	To identify how patients treated with medication for their depression perceived the relationship with their prescribing clinician
OKELLO 2007 [71]	Uganda	Female > Male	African	Secondary Care	Semi-structured Interview	Explanatory Model	Medical Records	A	To examine depressed patients’ perception of depression
PANG 1998 [72]	USA	Female > Male	Korean	Community	Semi-structured Interview	none	Diagnostic Interview	C	To explore the ways depression symptoms are expressed by elderly Korean women
ROGERS 2001 [73]	UK	Female > Male	not given	Primary Care	Semi-structured Interview	none	Medical Records	B	to explore experiences of depressed people with their contact with primary care
SARKISIAN 2003 [74]	USA	Male = Female	Mixed	Primary Care	Cross-sectional Survey	None	Screening Test	B	To determine whether older adults who attribute their depression to aging are less likely to believe seeking help is important
SCATTOLON 1999 [75]	Canada	Female	not given	Community	Semi-structured Interview	none	Self Diagnosis	A	Explore experiences of depression and their ways of coping
SHIN 2002 [76]	USA	Female > Male	Korean	Community	Semi-structured Interview & Focus Groups	none	Self Diagnosis	A	To investigate Korean Immigrants’ help-seeking behaviours for depression & under-utilization of mental health services
SRINIVASAN 2003 [77]	Canada	Female > Male	not given	Secondary Care	Cross-sectional Survey	None	Medical Records	B	The implications for patients perspectives for treatment preference, delivery & medication compliance
STECKER 2007 [78]	USA	Female > Male	Mixed	Primary Care	Cross-sectional Survey	None	Medical Records	B	To investigate whether attitudes towards psychotherapy in a population of primary care patients diagnosed with depression influenced the likelihood that they initiated psychotherapy
UGARRIZA 2002 [79]	USA	Female	not given	Secondary Care	Semi-structured Interview	Explanatory Model	Medical Records	B	What is the explanation of depression given by a group of older women with depression
VAN VOORHEES 2005 [80]	USA	Female > Male	Mixed	Online	Cross-sectional Survey	Theory of Reasoned Action	Screening Test	B	To develop a multivariate model of intent not to accept a diagnosis of depression
VAN VOORHEES 2006 [81]	USA	Female > Male	Mixed	Online	Cross-sectional Survey	Theory of Reasoned Action	Screening Test	B	Specifically, we examine the relationship between five types of actors and low self-perceived need for treatment: beliefs and attitudes towards treatment behaviors, subjective social norms, past treatment behaviors, illness factors, and personal characteristics.
WAGNER 1999 [82]	USA	Female > Male	Mixed	Community	Semi-structured Interview	none	Self Diagnosis	B	To examine the reasons people choose to self-medicate with St. John’s wort instead of seeking care from a conventional health care provider.
WAITE 2009 [83]	USA	Female	African American	Primary Care	Focus Group	Explanatory Model	Medical Records	A	To examine the explanatory models for depression among a cohort of low-income African American women
WILLIAMS 2001 [84]	UK	not given	not given	Primary Care	Semi-structured Interview	CS-SRM IR	Medical Records	A	To explore the perceptions of cause of psychological distress
WITTINK 2008 [85]	USA	Female > Male	Mixed	Primary Care	Semi-structured Interview	Cultural Models Theory	Medical Records	A	To identify health beliefs about depression in older adults and how they perceive differ from doctors
WITTKAMPF 2008 [86]	Netherlands	Equal	Mixed	Primary Care	Semi-structured Interview	none	Diagnostic Interview	B	To understand the views of patients’ who screened positive in a depression screening programme
YEUNG 2004 [87]	USA	Female > Male	Chinese	Primary Care	Cross-sectional Survey	Explanatory Model	Diagnostic Interview	C	To use the Explanatory Model Interview Catalogue to examine systematically the illness beliefs of depressed Chinese American patients seeking treatment at a primary care clinic.

**Figure 2:**
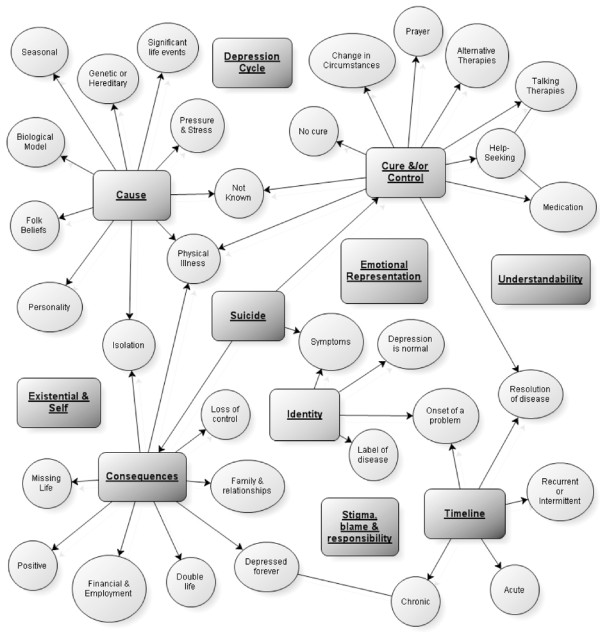
Map of illness beliefs and their corresponding subthemes.

Beliefs could be coded to all the main categories of illness representation. We developed five new thematic categories for beliefs that did not fit well into the illness representations framework. We labeled these: understandability; the depression cycle, existential and self, suicidal thinking and stigma, blame and responsibility. Table2 shows the studies that contributed to each theme. Figure2 shows themes with their associated subthemes.

**Table 2:** Table of themes identified in each included study

Study	Identity	Cause	Cure/Control	Consequences	Timeline	Understandability	Depression Cycle	Existential & Self	Suicide	Stigma, blame & responsibility
ADDIS 1195		✓								
ADDIS 1196		✓	✓							
ALLEN 1998	✓		✓							
AL-SAFFAR 2003		✓	✓	✓						
BACKENSTRASS 2007	✓	✓	✓		✓					✓
BADGER 2007b	✓	✓	✓		✓					
BANN 2004		✓	✓		✓					
BOGNER 2008	✓	✓	✓	✓		✓				
BROWN 2001	✓	✓	✓	✓				✓		✓
BROWN 2005			✓							
BROWN 2007	✓	✓	✓	✓	✓			✓		✓
BURROUGHS 2006	✓	✓	✓		✓	✓				✓
CABASSA 2008	✓	✓	✓	✓	✓					
CAPE 1999	✓	✓	✓		✓					✓
CHAKRABORTY 2009		✓	✓							
COOPER 1998	✓		✓							✓
COOPER 2000	✓		✓							✓
COOPER 2003			✓							✓
COOPER-PATRICK 1997	✓	✓	✓	✓						✓
CORNFORD 2007	✓	✓	✓	✓	✓		✓	✓		✓
DANIELSSON 2009	✓	✓	✓	✓	✓	✓		✓		✓
DEJMAN 2008	✓		✓							
EDLUND 2008	✓		✓							✓
FORTUNE 2004	✓	✓	✓	✓	✓					
GARFIELD 2003	✓	✓	✓	✓						✓
GASK 2003	✓		✓							✓
GIVENS 2006	✓	✓	✓		✓					✓
GIVENS 2007	✓	✓	✓	✓						✓
GREEN 2002	✓	✓	✓							✓
GRIME 2003	✓	✓	✓		✓					
HEIFNER 1997	✓	✓	✓	✓	✓					✓
KANGAS 2001	✓	✓	✓	✓	✓	✓	✓			✓
KARASZ 2003	✓	✓	✓	✓	✓					
KARASZ 2006	✓	✓	✓	✓	✓					
KARASZ 2008	✓	✓	✓	✓	✓					
KARASZ 2009	✓	✓	✓	✓	✓		✓	✓		
KARP 1994	✓	✓	✓	✓	✓			✓		✓
KELLY 2007	✓	✓	✓	✓	✓					
KIRK 2001	✓		✓	✓	✓					✓
KUYKEN 1992	✓	✓	✓							
LEWIS 1995	✓		✓		✓					✓
LEYKIN 2007		✓								
LOWE 2006	✓	✓	✓							
MANBER 2003	✓	✓	✓	✓	✓					
MARTIN 2007a	✓	✓	✓	✓	✓					✓
MARTIN 2007b	✓	✓	✓	✓	✓					
MAXWELL 2005	✓		✓	✓	✓					✓
NOLAN 2005	✓		✓	✓	✓					✓
OKELLO 2007	✓	✓	✓	✓	✓					✓
PANG 1998	✓	✓	✓	✓	✓					✓
ROGERS 2001	✓	✓	✓	✓	✓			✓		✓
SARKISIAN 2003		✓	✓							
SCATOLLON 1999	✓	✓	✓	✓	✓					✓
SHIN 2002	✓	✓	✓	✓	✓					✓
SRINIVASAN 2003		✓								
STECKER 2007			✓							
UGARRIZA 2002	✓	✓	✓	✓	✓					
VAN VOORHEES 2005	✓	✓	✓	✓						✓
VAN VOORHEES 2006		✓	✓							✓
WAGNER 1999	✓	✓	✓		✓					
WAITE 2009	✓	✓	✓	✓				✓		✓
WILLIAMS 2001	✓	✓	✓		✓					
WITTINK 2008	✓	✓	✓							
WITTKAMPF 2008	✓	✓	✓	✓	✓		✓			✓
YEUNG 2004	✓	✓	✓							✓

### Identity

Depression, depressed and depressive were the most commonly used labels by both authors and study participants. Other labels used by participants were stress, blues, nerves, sadness, loneliness and emotional or mental disorder.

Some participants said they would rather not know they had depression, whereas others believed the label meant treatment was possible and they were not ‘mad’. Some felt depression or its symptoms were a normal part of life and not a disease and that the word ‘depression’ is used in everyday language without meaning an illness.

*“It never occurred to me that I could be depressed, I just thought that I was a nasty person.”*[64]

*“Did I know what it was? It was pain, but I don’t think I would have called it depression. I think I would have called it my pain.”*[60]

### Cause

Most study participants could name at least one cause for their depression and many had complex, multi-factorial causes. Most participants believed the causes were external and took the form of significant negative life events and stress rather than subscribing to a mainly biological model. Co-existing physical illnesses were mentioned by several participants. Where the biological model was mentioned it was either to disagree with it or to mention it alongside other causal beliefs.

*“I have diabetes and other people with diabetes have experience with depression because of our treatment, and the things we have to do, and the way we have to live now [which is] different from the way we were used to doing things before.”*[59]

*“I think it [depression] is due to a lack of hormones, that is, a consequence of being exposed to stress over such a long period of time.”*[44]

### Cure and/or control

Study participants found it difficult to acknowledge the need for help; however, many believed the GP was the right person to approach. Participants had strong beliefs over whether medication or talking therapies would help them. Some mentioned beliefs about alternative therapies such as St. John’s wort or using prayer, often used alone if the depression was not seen as having a severe impact upon their life. A few participants were unable to identify any cure or control and some felt that depression is incurable.

*“When I talk about my problems I think about them and I feel worse. So I don’t know if it really is better to talk about them because you remember all your problems. Sometimes I feel better when I am doing other things and not talking about it to anyone.”*[58]

*“Well I think it’s a waste of time really, he’ll just give me yet another pill and I shall still be depressed because of all the other things that are wrong with me.”*[43]

### Timeline

The timescales participants mentioned for onset, duration and response to treatment were reported as varying markedly with acute, cyclical and chronic timescales being mentioned. The onset was described as “a bolt out of the blue” or “slow and insidious”. Treatment response was seen as a short or long process.

*“I am so afraid that I am going to remain depressed. That is the only thing I fear.”*[79]

*“Well, if it’s only something that’s going to be short term, then obviously it’s worth getting the help and then sort your problems out and see how things go after that, more or less.”*[69]

### Consequences

Depression was seen as having mostly negative consequences, affecting all present and future aspects of life, including work, social and home life and physical health, especially where there was a co-existing illness. Some held particular fears of losing control and embarrassing themselves.

*“So you’ll be dying of sadness, you get that sadness because the doctors say that if you stay really sad you begin to get other types of diseases like those that come from anguish, sadness, from depression you go on getting other types of disease and you end up dying too. Besides depression, it sets off other systems within your organism and ends up killing you.”*[88]

*“I was already on a pedestal, being the first doctor ever in the family, and my mom and dad didn’t want this to take me down from that pedestal in the other family’s eyes.”*[54]

### Emotions

We could not distinguish an emotional representation for depression from emotional symptoms of depression. Participants associated depression emotionally with fear, anger, sadness, despair, and guilt.

*“Anxiety, anger, confusion, frustration for me is associated with the depression. Not sadness so much.”*[59]

*“I’m afraid…of being an invalid…not doing the things I want to do.”*[32]

### Understandability

Participants’ beliefs about depression were not always internally consistent. Some understood their depression in terms of their life story and gave coherent beliefs. Thereby, a woman who believed the cause of her depression was her poor eyesight that stopped her from doing things believed the cure for this was to “get her eyes sorted” [35]. For others depression was “unexplained” and “not understood” which led to conflicting and less fixed beliefs. For example, one study reported of people with depression “Their explanations changed within their narratives and they tried out several explanations, not finding one that explained all of their experience” [55], leading to uncertainty about how to resolve problems.

*Sometimes the account of depression contained several narrative episodes based on more than one storyline.*[55]

### Depression cycle

Depression was sometimes seen as a spiraling process, with episodes being both a consequence of previous depression and a cause of new onset of depression. These data could not be coded to the cause construct or the cyclical timeframe construct as one episode of depression was believed to be the cause of a subsequent episode of depression itself. Being depressed caused individuals to become more isolated and lonely, and made the sufferer further depressed. Having depression left the person with an internal weakness and predisposed to future depression, a cycle from which it was hard to break out. There are few other diseases where the disease itself can be seen in this way, and in this respect, this depression belief is unique.

*“Anxieties cause depression and depression causes self-depreciation.”*[59]

### Existential and self

This theme concerned the individual’s sense of identity and differs from the identity of the disease theme. For some participants, even more than in physical diseases, depression is deeply interwoven in everyday life, in an existential understanding of the self and in a person’s sense of social and individual identity. It defined the person as who they were in their entirety, not as a consequence of depression but more of a statement of their individuality. Having depression changed the way they viewed themselves and their personality. Depression gave them a new identity, and they joined a category of person in which they had not previously seen themselves. For many this was a distressing and unwelcome experience. On questioning, many participants strongly agreed that having depression affected the way they saw themselves as a person [24,34].

*“You know, I was a mental patient. That was my identity…depression is very private…It’s no longer just my own pain. I am a mental patient. I am a depressive. I am a depressive [said slowly and with intensity]. This is my identity. I can’t separate myself from that. When people know me they’ll have to know about my psychiatric history, because that’s who I am.”*[60]

*“And when I came out I did feel quite odd because she gave me a prescription. I couldn’t. I suddenly felt like I fell into a bracket of a type of people, emotionally in my head. Which is quite a strange feeling really because . . . I’m not like I thought I was and now I’m a bit different”*[48]*.*

### Suicidal thinking

Suicide was rarely mentioned but when it was, it had an ambiguous status and did not fit within an obvious theme and was often a gender specific belief. Suicidal thinking was seen as symptom of depression, something people would never suffer with when well. For others, suicide was a consequence; the depression was so severe that suicide was an understandable response to suffering. It could also be seen as a control mechanism – a means of getting relief from their distress, and the most acceptable way of dealing with a problem. Suicide required a lot of self control and counteracted the image of being weak for having depression, particularly amongst males [54].

*“In the beginning, you may not know what’s happening to you… if it gets worse and you don’t get help, people eventually hurt themselves with drugs or they can take their own life.”*[83]

*“Men who kill themselves are doing what maybe a lot of men have been taught to do. Literally they are taking their lives in their own hands because that’s what guys are taught to do. You know, to take care of it, and they take care of it in a way that is absolutely what they believe to be the right thing to do.”*[54]

### Stigma, blame and responsibility

Participants feared the outcome of others knowing about their condition. Depression was seen as poorly understood by the public, and misrepresented in the media, so that sufferers were to blame or responsible for their depression. This idea of blameworthiness was different from the situation when external factors were clearly contributing. For example, a severe economic depression in Finland was seen as being responsible for the increase in depression and was socially acceptable [55]. Participants were ashamed of being seen as not been able to cope – the stigma beliefs they had attached to people suffering from depression – and were now a part of themselves. Perceived stigma in itself had consequences, such as their judgment would no longer be trusted whilst they suffered from depression, leading to employment problems and the loss of friendships.

*“When you have an operation you have friends who you can talk to. Last year, I had an accident and I received 45 get-well cards, but you go down with depression and nobody knocks on your door.”*[70]

*“The reason why it was hard to get psychiatric help was because of pride. I didn’t want people to think, “How did he end up this bad?” I just don’t want to be one of those crazy people, and it’s basically admitting that I am not in charge of my own emotions.”*[76]

## Discussion

### Summary of main findings

Our most striking finding is the wide range of beliefs held by people experiencing symptoms when they are questioned, and importantly, although we started with a framework based upon how people think about illness, not all the beliefs we identified could be fitted into this structure. These themes have not been actively looked for in previous literature and therefore the number of studies contributing to each theme is small. They could not be fitted into the illness representations framework without losing some of the most interesting and potentially clinically significant beliefs about depression.

Our new themes support ideas from the health psychology literature. Sense of Coherence is said to assist individual coping with illness by facilitating understanding of the challenge of illness and by allowing the individual to integrate the illness experience with a sense of personal meaningfulness [89]. It is therefore closely related to our themes of understandability and existential and self. Sense of Coherence has been associated with good health and especially with good mental health [90].

There was no evidence that the selection criteria and depression status of the participants influenced beliefs. For example beliefs about whether depression symptoms are a normal part of life were endorsed in studies including participants who were recruited following self-diagnosis of depression, a diagnosis in the medical records and those identified by screening or diagnostic interview. This suggests that beliefs about depression can be similar, regardless of whether a person is formally diagnosed.

### Comparison with existing literature

We identified a greater diversity of beliefs than in previous review of the beliefs of people with current depression [16], perhaps because we actively looked for beliefs outside the framework of illness representations. That study also had a wider focus, including beliefs of the general public and those suffering from other problems such as anxiety [16].

### Strengths and limitations of the study

Our review strengths include the comprehensive search strategy, the development of themes from methodologically robust studies, the systematic approach to synthesis and the integration of both qualitative and quantitative data. We used one increasingly common approach to integrating mixed data although others exist [19]. The sensitive search strategy employed meant that many non-relevant articles were found in the searches; however this has hopefully ensured that no relevant articles were missed.

Stage 1 identified only two studies with beliefs associated with a chronic physical illness [32,79]. So in stage 2 we included all depression beliefs. Beliefs associated with a physical illness may differ from those which are not, but the difference is likely to be on emphasis rather than in specific content. For example, symptoms of illness or its treatment may be seen as a cause of depression; or physical symptoms and depression may interact so that the consequences of their co-existence are felt more severely. Cause and consequence are existing themes in our framework, here given new content but not displaced by a new theme. Limiting the scope of this review to primary care may have meant that potentially relevant studies were missed but increased the relevance of this review to the current management of depression in primary care, such as case-finding for at-risk people. In the absence of established methodological consensus on whether or not to include quotations from original studies in a review of this type, we opted for inclusion to enhance illustration of the themes [91].

### Implications for further research and clinical practice

As evidence accumulates [92] to show that chronic physical disease is a risk factor for depression, and that depression has a detrimental effect on morbidity and mortality, health professionals are likely to be encouraged to actively seek such at risk people. If we are unable to understand how patients think about depression and take into account their beliefs then the uptake and outcomes of depression screening are likely to be compromised, as well as patient concordance with any subsequent depression management. Particularly important are likely to be beliefs about the inappropriateness of having a quasi-diagnostic label, about the origins of depression in life problems and about medication being inappropriate. Equally important but often neglected is the evidence that not everybody thinks of depression as being illness-like, such beliefs being incompatible in a more fundamental way with interventions based upon screen-treat approaches in healthcare. Our findings are relevant to patients with physical illnesses and we are undertaking two further studies to investigate beliefs about depression associated with a physical illness to pursue this. In Table3 we summarize the clinical implications of our findings.

**Table 3:** Implications derived from themes

Theme	Implications
Identity	How patients think about depression and about being given a label or diagnosis for it may be important in understanding why patients engage or do not engage in detection.
Cause	Mismatches in what patients and GPs believe causes depression may undermine the development of shared treatment plans and undermine subsequent concordance.
Cure &/or Control	Beliefs about the role and relevance of antidepressants or psychotherapy may affect whether patients wish to have depressive symptoms detected.
Timeline	Patient beliefs about the course of their depression will affect detection. Those who expect quick resolution may not think it to be appropriate to seek treatment.
Consequences	Negative views about the consequences of having depression may lead to hopelessness or defensiveness in the face of attempts at standardised depression detection.
Coherence	Identifying how the patient thinks can be difficult in consultations, but it will be important to identify and if possible moderate beliefs if they are not helpful to recovery.
Depression Cycle	The cyclical beliefs leave patients feeling a sense of futility about long term approaches to intervention.
Existential & Self	Discussing what depression means to how patients perceive themselves may increase acceptance by a patient that depression can be a concern of clinicians.
Role of suicide	While suicidal acts are relatively rare, suicidal thoughts are relatively common. Exploring the latter is best with an open mind towards their meaning for the patient.
Stigma, blame & responsibility	Presenting screening as a normal and routine part of care may help reduce feelings of shame and “give permission” to discuss depression.

These considerations are important when there is a financial reward for administering a screening test without clear evidence of benefit, and when patients do not undergo an informed consent process which includes the risks and benefits of testing prior to the test being administered.

## Conclusions

We need approaches to detection of depression in physical illness that are sensitive to the range of beliefs held by patients. Further research is needed to understand fully how people comprehend depression associated with a physical illness and how this influences help-seeking and engagement with health care services.

## Appendix 1 – search terms

### A.1. Ovid MEDLINE (1950-present day)

1) exp *Attitude to Health/

2) exp *Health Knowledge, Attitudes, Practice/

3) (illness adj2 (cognit* or schemat* or percept* or represent* or belie* or attitud* or behav* or reason*)).tw.

4) (depress* adj2 (cognit* or schemat* or percept* or represent* or belie* or attitud* or behav* or reason*)).tw.

5) exp *“Patient Acceptance of Health Care”/

6) exp *Models, Psychological/

7) (health belie* adj2 model*).tw.

8) (theor* adj2 plan* adj2 behav*).tw.

9) (health* adj2 action* adj2 process*).tw.

10) (social* adj2 cognit* adj2 model*).tw.

11) (protect* adj2 motiv* adj2 theor*).tw.

12) (theor* adj2 reason* adj2 action*).tw.

13) (common* adj2 sense*).tw. 1676

14) (self* adj2 regulat*).tw.

15) 1 or 2 or 3 or 4 or 5 or 6 or 7 or 8 or 9 or 10 or 11 or 12 or 13 or 14

16) exp *Depression/

17) exp *Depressive Disorder/

18) depress*.tw.

19) 16 or 17 or 18

20) exp Family Practice/

21) exp Primary Health Care/

22) exp Physicians, Family/

23) ((general or family) adj practi$).tw.

24) family physic$.tw.

25) (primary adj2 care).tw.

26) (gp or gps).tw.

27) 20 or 21 or 22 or 23 or 24 or 25

28) 15 and 19 and 27

29) limit 28 to “all adult (19 plus years)”

### A.2. Ovid Embase (1980-present day)

1) exp *attitude to health/

2) exp *health belief/

3) exp *Health Belief Model/

4) exp *patient attitude/

5) (illness adj2 (cognit* or schemat* or percept* or represent* or belie* or attitud* or behave* or reason*)).tw.

6) (depress* adj2 (cognit* or schemat* or percept* or represent* or belie* or attitud* or behave* or reason*)).tw.

7) exp psychological model/

8) (health belie* adj2 model*).tw.

9) (theor* adj2 plan* adj2 behav*).tw.

10) (health* adj2 action* adj2 process*).tw.

11) (social* adj2 cognit* adj2 model*).tw.

12) (protect* adj2 motivat* adj2 theor*).tw.

13) (theor* adj2 reason* adj2 action*).tw.

14) (common* adj2 sense*).tw.

15) (self* adj2 regulat*).tw.

16) 1 or 2 or 3 or 4 or 5 or 6 or 7 or 8 or 9 or 10 or 11 or 12 or 13 or 14 or 15

17) exp *depression/

18) depress*.tw.

19) 17 or 18

20) exp general practice/

21) exp primary health care/

22) exp general practitioner/

23) ((general or family) adj pract*).tw.

24) family physic*.tw.

25) (primary adj2 care).tw.

26) (gp or gps).tw.

27) 20 or 21 or 22 or 23 or 24 or 25 or 26

28) 16 and 19 and 27

### A.3. Ovid PsychINFO (1806-present day)

1) exp *Client Attitudes/

2) exp *Consumer Attitudes/

3) exp *Health Attitudes/

4) exp *Health Knowledge/

5) exp *“Mental Illness (Attitudes Toward)”/

6) (illness adj2 (cognit* or schemat* or percept* or represent* or belie* or attitud* or behave* or reason*)).tw.

7) (depress* adj2 (cognit* or schemat* or percept* or represent* or belie* or attitud* or behave* or reason*)).tw.

8) (health belie* adj2 model*).tw.

9) (theor* adj2 plan* adj2 behav*).tw.

10) (health* adj2 action* adj2 process*).tw.

11) (social* adj2 cognit* adj2 model*).tw.

12) (protect* adj2 motiv* adj2 theor*).tw.

13) (theor* adj2 reason* adj2 action*).tw.

14) (common* adj2 sense*).tw.

15) (self* adj2 regulat*).tw.

16) 1 or 2 or 3 or 4 or 5 or 6 or 7 or 8 or 9 or 10 or 11 or 12 or 13 or 14 or 15

17) exp *major depression/

18) exp *“Depression (Emotion)”/

19) depress*.tw.

20) 17 or 18 or 19

21) exp Primary Health Care/

22) exp General Practitioners/

23) exp Family Medicine/

24) exp Family Physicians/

25) ((general or family) adj practi*).tw.

26) family physic*.tw.

27) (primary adj2 care).tw.

28) (gp or gps).tw.

29) 21 or 22 or 23 or 24 or 25 or 26 or 27

30) 16 and 20 and 29

31) limit 30 to adulthood <18+ years>

### A.4. EBSCO CINAHL (1982-present day)

1) (MM “Attitude to Mental Illness”)

2) MM “Patient Attitudes”

3) MM Attitude to health

4) TX (health belie* N2 model*) or TX (theor* N2 plan* N2 behav*) or TX (health* N2 action* N2 process*) or TX (social* N2 cognit* N2 model*) or TX (protect* N2 motiv* N2 theor*) or TX (theor* N2 reason* N2 action*) or TX (common* N2 sense*) OR TX (self* N2 regulat*)

5) TX illness N2 cognit* or schemat* or percept* or represent* or belie* of attitud* or behav* or reason*

6) TX depress* N2 cognit* or schemat* or percept* or represent* or belie* or attitud* or behav* or reason*

7) 1 or 2 or 3 or 4 or 5 or 6

8) (MM “Depression+”)

9) TX depress*

10) 8 or 9

11) (MH “Family Practice”)

12) (MH “Primary Health Care”)

13) (MH “Physicians, Family”)

14) TX (general or family N2 practi*) or TX family physic* or TX primary N2 care or TX (gp or gps)

15) 11 or 12 or 13 or 14

16) 7 and 10 and 15

### A.5. ISI web of science (including science citation index expanded, and conference proceedings citation index, 1898–present day)

1) Topic = (attitude to mental illness)

2) Topic = (patient attitudes)

3) Topic = (attitude to health)

4) Topic = (health knowledge)

5) Topic = (patient acceptance of healthcare)

6) Topic = ((illness SAME (cognit* OR schemat* OR percept* OR represent* OR belie* OR attitud* OR behav* or reason*))) OR Topic = ((depress* SAME (cognit* OR schemat* OR percept* OR represent* OR belie* OR attitud* OR behav* or reason*)))

7) Topic = (psychological models) OR Topic = ((health belie* SAME model*)) OR Topic = ((theor* SAME plan* SAME behave*)) OR TOPIC = ((health SAME action* SAME process*)) OR Topic = (((social* SAME cognit* SAME model*)) OR Topic = ((protect* SAME motiv* SAME theor*)) OR Topic = ((theor* SAME reason* SAME action*)) OR Topic = ((common* SAME sense*)) OR Topic = ((self* SAME regulat*))

8) 1 OR 2 OR 3 OR 4 OR 5 OR 6 OR 7

9) Topic = (depression) OR Topic = (depress*)

10) Topic = (primary SAME care) OR Topic = (Family SAME physic*) OR Topic = (gp OR gps) OR Topic = ((general OR family) practice*) OR Topic = (family SAME medic*)

11) 8 AND 9 AND 10

### A.6. Cochrane library, Wiley InterScience, 2009 issue 4 (including the Cochrane database of systematic reviews, database of abstracts of reviews of effects, Cochrane central register of controlled trials, health technology assessment database, NHS economic evaluation database, and about the Cochrane collaboration)

1) Title – depress* AND (attitude* OR belie* OR percept* OR cognit* OR schemat* OR represent* OR behave* OR reason*)

### BIOSIS (previews)

1) exp Behavioral biology - Human behavior/

2) (illness adj2 (cognit* or schemat* or percept* or represent* or belie* or attitud* or behav* or reason*)).tw.

3) (depress* adj2 (cognit* or schemat* or percept* or represent* or belie* or attitud* or behav* or reason*)).tw.

4) (health belie* adj2 model*).tw.

5) (theor* adj2 plan* adj2 behav*).tw.

6) (health* adj2 action* adj2 process*).tw.

7) (social adj2 cognit* adj2 theor*).tw.

8) (protect* adj2 motiv* adj2 theor*).tw.

9) (theor* adj2 reason* adj2 action*).tw.

10) (common* adj2 sense*).tw.

11) (self* adj2 regulat*).tw.

12) 1 or 2 or 3 or 4 or 5 or 6 or 7 or 8 or 9 or 10 or 11

13) exp “Behavioral and Mental Disorders”/

14) exp Psychiatry/

15) depress*.tw.

16) 13 or 14 or 15

17) ((general or family) adj practi*).tw.

18) family physic*.tw.

19) (primary adj2 care).tw.

20) (gp or gps).tw.

21) 17 or 18 or 19 or 20

22) 12 and 16 and 21

23) limit 22 to human

24) limit 23 to adult

### A.7. The National Institute for Health Research Clinical Research Network Coordinating Centre (NIHR CRN CC)

1) Topic – All

2) Title – depression

### A.8. The national research register archive

1) Keywords – depressive disorder AND attitude-to-health

### A.9. Www.ClinicalTrials.Gov

1) Title – depression AND attitudes

### A.10. OpenSIGLE – grey literature

1) Keyword = depression

## Competing interests

All authors have completed the Unified Competing Interest form at http://www.icmje.org/coi_disclosure.pdf (available on request from the corresponding author) and declare: no support from any organisation for the submitted work; no financial relationships with any organisations that might have an interest in the submitted work in the previous three years, no other relationships or activities that could appear to have influenced the submitted work.

## Authors’ contributions

AH was responsible for the study conception and design, contributed to the interpretation of the data. RF, LG AH and KM commented on drafts of the manuscript. SA wrote the protocol and was responsible for data extraction, analysis and interpretation, drafting the manuscript and incorporating comments. RF and LG contributed to the interpretation of the data. KM acted as a second reviewer. SA will act as guarantor. All authors read and approved the final manuscript.

## Funding

This project was not externally funded.

## Pre-publication history

The pre-publication history for this paper can be accessed here:

http://www.biomedcentral.com/1471-2296/13/41/prepub
